# The Impact of MicroRNAs on Brain Aging and Neurodegeneration

**DOI:** 10.1155/2012/359369

**Published:** 2012-01-18

**Authors:** Stephan P. Persengiev, Ivanela I. Kondova, Ronald E. Bontrop

**Affiliations:** ^1^Department of Complex Genetics, Division of Medical Genetics, University Medical Center Utrecht, 3584 CG Utrecht, The Netherlands; ^2^Animal Science Department, Biomedical Primate Research Center, Lange Kleiweg 139, 2288 GH Rijswijk, The Netherlands; ^3^Department of Comparative Genetics and Refinement, Biomedical Primate Research Center, Lange Kleiweg 139, 2288 GH Rijswijk, The Netherlands

## Abstract

The molecular instructions that govern gene expression regulation are encoded in the genome and ultimately determine the morphology and functional specifications of the human brain. As a consequence, changes in gene expression levels might be directly related to the functional decline associated with brain aging. Small noncoding RNAs, including miRNAs, comprise a group of regulatory molecules that modulate the expression of hundred of genes which play important roles in brain metabolism. Recent comparative studies in humans and nonhuman primates revealed that miRNAs regulate multiple pathways and interconnected signaling cascades that are the basis for the cognitive decline and neurodegenerative disorders during aging. Identifying the roles of miRNAs and their target genes in model organisms combined with system-level studies of the brain would provide more comprehensive understanding of the molecular basis of brain deterioration during the aging process.

## 1. The Aging Profile of the Human and Nonhuman Primate Brain

The molecular and structural transformations that shape the human cognitive abilities occur mostly in the period between birth and adulthood although some developmental processes, such as cortical axon myelinization, extend beyond this time window [1–3]. The primate brain is subjected to dramatic change, both structurally and functionally, during postnatal development [1, 4]. It is quite remarkable that the process of brain aging begins at early adulthood that is manifested by gradual deterioration of the brain capacity to utilize the flow of information. In later life, the brain begins to change in a more destructive manner. Such changes include a decrease in brain volume, loss of synapses, cognitive decline, and a rise in the frequency of neurological disorders [2, 5–7]. Although developmental and aging-related changes are clearly observed histologically and in cognitive function, their molecular underpinnings are still poorly understood.

Multiple cellular and functional transformations take place in the brain during aging. Neural cells may respond to these changes by reprogramming metabolic circuits in order to adapt and maintain its functionality, or they may give in to neurodegenerative cascades that result in disorders such as Alzheimer's, cerebellar ataxias, and Parkinson's diseases. A number of mechanisms are employed to maintain the integrity of nerve cell networks and to facilitate responses to external and internal environmental stimuli and maintain neuron integrity and functional capability after damage. The protective machinery includes production of neurotrophic factors and cytokines, expression of various cell survival-promoting proteins (e.g., antioxidant factors, prosurvival and antiapoptotic proteins, and protein chaperones), activation of DNA caretaker cascades to preserve the genomic integrity, and mobilization of neural stem cells to replace damaged neurons and glial cells. The aging process presents a challenge for the neuroprotective and neurorestorative mechanisms. Genetic background and environmental stressors superimposed upon the aging dynamic are the determining factors of the physiological versus pathological brain aging. The importance of genetic predisposition to accelerated aging and neurodegeneration is well documented. The accumulation of toxic proteins transcribed from mutated genes causes inherited forms of Alzheimer's disease (amyloid precursor protein and presenilins), Parkinson's disease (*α*-synuclein and Parkin), and trinucleotide repeat disorders (huntingtin, androgen receptor, ataxin, and others) by overcoming the endogenous neuroprotective mechanisms. The neuroprotective mechanisms can be augmented by dietary and behavioral modifications, such as caloric restriction, antioxidant supplements, and physical activities. Also, activating a response in which neurons increase production of neurotrophic factors and stress proteins can facilitate physiologically adequate brain aging. Neural stem cells that reside in the adult brain are also responsive to environmental demands and appear capable of replacing lost or dysfunctional neurons and glial cells revealing a remarkable capacity within brain cells for adaptation to aging and resistance to disease.

Although rodent models to study brain aging and neurodegenerative disorders have been developed, these models do not satisfactorily parallel the brain changes and behavioural features observed in humans. The close physiological, neurological, and genetic similarities between humans and higher primates offer the opportunity to study the aging process and associated abnormalities in monkeys [8, 9]. Aged nonhuman primates undergo age-associated structural and functional brain changes similar to those that occur in aged humans and, to some degree, in individuals with Alzheimer's disease [9]. As in humans, declines in performance on cognitive and memory tasks begin at the monkey equivalent of late-middle life. The brains of old monkeys show degenerative changes in neurons, abnormal axons and neurites, and accumulations of amyloid plaques and lipofuscin around blood vessels and in the residential macrophages [8]. Moreover, old non-human primates exhibit decline of specific neurotransmitter networks, most notably the forebrain cholinergic system that has been suggested to contribute to the memory deficit characteristic for older individuals.

## 2. Brain Aging and MicroRNA Expression Profiles

Genome-wide gene expression studies during ageing of the nematode *Caenorhabditis elegans*, the common fruit fly (*Drosophila melanogaster*), and the brains of mice, rats, chimpanzees, and humans have revealed a few broadly conserved functional categories of genes with age-dependent expression changes [17–23]. In particular, most of these studies provided evidence of reduced mitochondrial function and increased expression of stress-response genes during aging that were even more pronounced in humans with cognitive decline and neurodegenerative disorders [24]. Thus, it appears that some conserved mechanisms of stress resistance are activated during the brain aging to protect against the pathology of neurodegenerative disorders. However, despite clear evidence of conservation of ageing pathways and gene expression signatures, the direct comparison of gene expression during ageing in mouse, rhesus macaque, and human brain has revealed a major evolutionary divergence [19].

Factors such as DNA methylation, histone modification, chromatin remodeling, and small noncoding RNAs can all contribute to the broad variety of phenotypes of ageing. Among these, the group of small noncoding RNAs called microRNAs (miRNAs) have been recently found to have significant impact on brain aging as well as cellular senescence [25–27]. In the past decade, the accumulated knowledge of small noncoding RNAs (ncRNAs) has provided new understanding in the spatiotemporal regulation of gene expression. The category of small noncoding regulatory RNAs include miRNAs, small interfering RNAs (siRNAs), PIWI-interacting RNAs (piRNAs), small nucleolar RNAs (snoRNAs), and small nuclear RNAs (snRNAs). Thus far, the best studied small ncRNAs are the microRNAs (miRNAs), a large group of short (18–25 nucleotide), noncoding RNA molecules transcribed either by RNA polymerase II or RNA polymerase III. Most miRNA-mediated regulation occurs at the posttranslational level, primarily through its near-perfect or partial complementary to consensus elements within 3′-untranslated region (3′ UTR) of target mRNA, leading to translational repression and/or degradation. In some cases, they may also promote translation [28, 29] (Persengiev, unpublished data).

miRNAs play an important role in the regulation of several cell processes, including cell proliferation, development, cancer formation, stress responses, and apoptosis. The rapid progression of miRNA research in these areas has revealed its prominent role in modulating gene expression. However, the role of miRNAs in senescence remains poorly understood. miRNA can affect pathways involved in ageing, and miRNA profiling has shown significant alterations in their expression level. Importantly, recent data have shown the significance of miRNA in brain aging and neurodegeneration [26, 27, 30, 31]. The genome-wide expression analysis of miRNAs in aging individuals revealed a general decline in miRNA levels that was linked to potential loss of control of cancer-associated genes [32]. Nine miRNAs (miR-103, miR-107, miR-128, miR-130a, mIR-155, miR-24, miR-221, miR-496, and mIR-1538) were identified to be significantly lower in the peripheral blood mononuclear cells of old individuals as compared to the young subjects that were identified in this study.

Identifying the regulatory circuitry processes that control cell differentiation and transmission of information between neurons is fundamental to understanding changes in the aging brain. miRNAs regulate expression of protein-coding genes [33, 34]. Several lines of evidence indicate that miRNAs contribute to the control of brain development and its functional and structural reorganization, as a result of age progression and deterioration of neuronal metabolism. A subset of miRNAs is selectively expressed in brain tissues [35], and miRNA expression profiling in the adult brain of different species separated the human brain regions from those of chimpanzee, mouse, and rat [19, 36, 37]. Moreover, targeted inactivation of Dicer miRNA processing endonuclease was found to lead to degeneration of Purkinje cells [34] and retinal cells deficient for Dicer undergoes a progressive degeneration [33]. Specific miRNAs have been shown and in some cases predicted with high confidence to be involved in Alzheimer's disease, spinocerebellar ataxia type 1, Parkinson's disease, and amyotrophic lateral sclerosis (ALS), in addition to the general dysregulation of miRNA expression observed in neurodegenerative disorders [10–16, 26, 38–40] (see Table 1 for details). However, how miRNA expression is regulated during brain aging and how miRNAs participate in the regulatory loops controlling brain function is not well understood.

**Figure 1:**
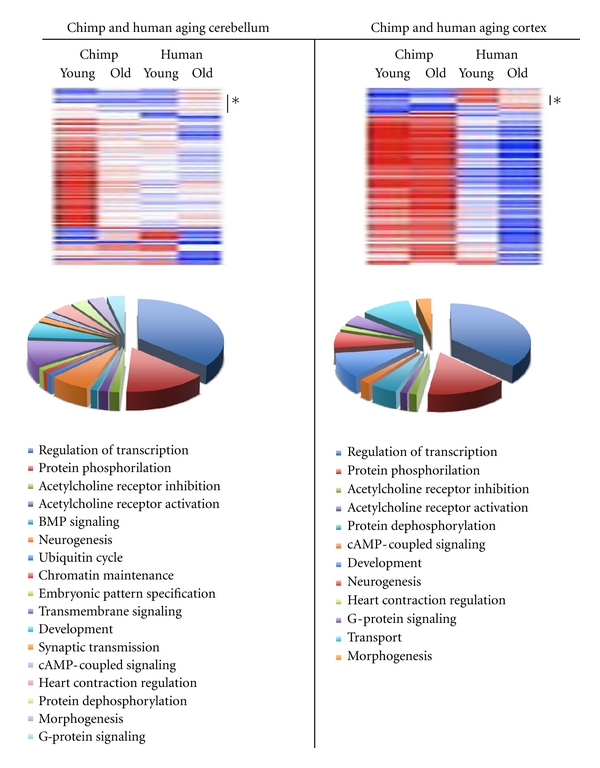
Expression of selected miRNAs is induced in the aging cerebellum and cortex of chimpanzees and humans. The asterisks indicate groups of miRNAs and ncRNAs that are specifically upregulated in the aging human cerebellum and cortex. Ontology analysis for biological function of the human upregulated miRNA target genes in the cerebellum and cortex of aged individuals is shown below the heatmaps.

We recently performed a genome-wide expression screen of miRNAs and ncRNAs in the brain of chimpanzees and humans and found that miRNA expression is differentially regulated in the cortex and cerebellum of humans and non-human primates during aging. The miRNA levels remain relatively stable in the cortex in contrast to the general miRNA downregulation associated with the aging cerebellum. This observation is significant and apparently reflects the temporal functional status of neuronal activity in the cortex and cerebellum. However, there was no unifying specific miRNA pattern associated with the brain aging. Despite this observation, the group of targeted genes was remarkably conserved during evolution especially when their role in biological process regulation was used as criteria for the ontological analysis (Figure 1). Interestingly, the data identified miR-144 to be the sole miRNA that was consistently upregulated in the aging chimp and human cerebellum and cortex [13]. The selective increase of miR-144 levels suggests that miR-144 is likely to play a coordinating role in the posttranscriptional regulation of a group of genes that are subjected to strong miRNA control in the aging brain. This finding implies that the onset of aging, responsiveness to environmental stress signals, and the associated risk of disease development are encoded within the genes themselves which in turn determines the individual gene expression profiles.

## 3. The Adaptive Role of MicroRNAs in Neurodegeneration

The brain is a complex organ, with various types of neurons and nonneuronal cell types that form an intricate communication network. Aging-related neurodegenerative diseases are the culmination of many different genetic and environmental influences. Prior studies have shown that RNA regulation is altered during the course of some neurodegenerative disorders. Recently, a number of papers suggested that miRNAs might be a contributing factor in neurodegeneration. The experimental data have shown that many miRNAs are expressed in specific brain regions, suggesting their functional role in brain activities. For instance, miRNAs have been implicated in neural cell developments, and miR-128 was shown to reduce the expression of the neural stem cell renewal factor Bmi-1 [41]. In the mammalian brain, miR-9 and miR-132 are expressed in hippocampus and medal frontal gyrus [26]. miR-9 expression is necessary for neurogenesis in cultured stem cells and its downregulation correlates with premature cortical differentiation in presentiling-1 knockout mice [42]. miR-132 is an inhibitor of p250GAP translation that plays a role in neurite extension and neurogenesis [43]. In addition, miR-132 has been linked to BDNF, a member of the nerve growth factor family that is necessary for survival of striatal neurons in the brain, and MeCP2 methyl-CpG DNA binding protein that plays an essential role in mammalian development [44]. Another miRNA, miR-124a, plays a role in the differentiation of neuronal progenitors into mature neurons by controlling the elimination on nonessential transcripts [45]. miR-9, miR-132, and miR-124a expression is inhibited in nonneural cells by the transcriptional repressor REST that helps to preserve the identity of nervous tissue by silencing neuronal genes in nonneural tissues [46].

Research about miRNAs in the context of neurodegeneration is accumulating rapidly, and recently published studies suggested a role of miRNAs in Alzheimer's disease, Parkinson's disease, and triplet repeat disorders. In the cortex of Alzheimer patients, the expression of miR-107 was reduced significantly even in patients with very early pathological alterations [47]. miR-107 lowers the expression of b-site amyloid precursor protein-cleaving enzyme 1 (BACE1) and may be involved in the acceleration of Alzheimer's disease progression. Disruption of miR-433 binding site in fibroblast growth factor 20 (FGF20) 3′ UTR correlates with increased alfa-synuclein expression in Parkinson patients [48]. miR-133b that is specifically expressed in the midbrain dopaminergic neurons and functions to fine-tune the dopaminergic behaviours, such as locomotion, is deficient in midbrain tissues from Parkinson patients [49]. Thus, a connection between miRNA and ageing-associated neurodegenerative abnormalities is emerging and provides new insights into the mechanisms of disease development.

**Figure 2:**
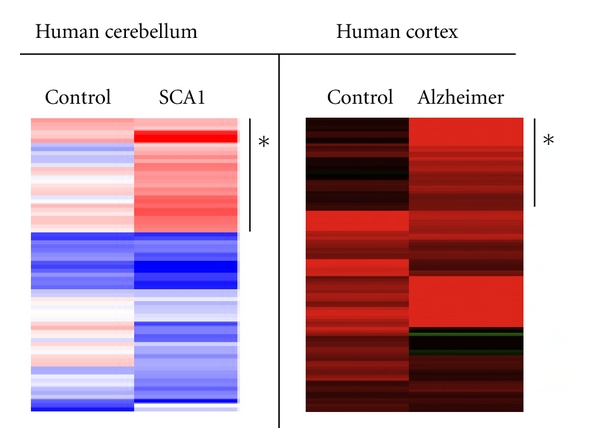
Activation of miRNA expression in the affected brain compartments of Alzheimer's and SCA1 patients. The asterisks indicate the subsets of miRNAs and ncRNAs that are specifically upregulated in SCA1 cerebellum and Alzheimer's cortex.

miRNAs are generally considered to play defensive functions at the level of the whole organism [27, 34, 50, 51]. We observed a general induction of miRNA and ncRNA expression in the brain compartments of SCA1 and Alzheimer patients (Figure 2). It is expected that downregulation of miRNA expression during the progress of aging would lead to a diminished control on posttranscriptional processing of gene expression. The higher levels of processed miRNA and ncRNA transcripts in the cerebellum and cortex diagnosed with neurodegenerative disorders might be a compensatory reaction to diminish the effect of aberrant proteins buildup in the affected compartments. Two different mechanisms may account for this phenomenon: first, miRNA response might be directly or indirectly linked to the mutant protein expression (e.g., ATXN1 in spinocerebellar ataxia type 1 and APP in Alzheimer's disease), or second, a result of a general transcriptional deregulation in the affected tissues. The aging brain retains the expression of small number of miRNAs that are able to exercise a limited but adequate control on gene expression and support neuron functioning. miRNA expression is markedly increased in the brain of SCA1 and Alzheimer patients that suggests that the trigger for the miRNA response is the excessive amount of toxic metabolites and is mainly directed to restrict their negative effect. However, it is worth considering that the overactivation of miRNA machinery, as the process of neuron degeneration progresses, could have a negative impact on the neuron function. Thus, a detailed understanding of the mechanisms and factors involved in the miRNA processing in the brain during normal aging and in aging-associated pathologies that are manifested by progressive loss of function will require further studies.

A long-standing question in the field of brain aging and neurodegeneration is how the ubiquitous expression of mutant proteins can be reconciled with their neuron-specific cytotoxic effect in defined brain compartments. For instance, it is well documented that the expanded polyglutamine proteins, such as ATXN1, are ubiquitously expressed, but the disease affects only subsets of neurons. These data suggest that the sensitivity of particular neuron population to the mutant protein is facilitated by additional mechanism(s) and might incorporate the effectiveness of miRNA response. Thus, it is likely that the maintenance of selected miRNA expression in the aging brain serves as a default protective function, and the deregulation of this mechanism contributes to neurodegenerative disorders.

## 4. Regulatory Pathways Modulated by MicroRNAs in the Brain

### 4.1. Apoptosis and Cell Survival Pathways

Recent research has demonstrated that miRNAs are key regulators of cell death mechanism acting either as inhibitors of apoptosis activation or proapoptotic factors. Many miRNAs are antiapoptotic and mediate this effect by targeting pro-apoptotic mRNAs or positive regulators of pro-apoptotic mRNAs. On the other hand, pro-apoptotic miRNAs target anti-apoptotic mRNAs or their positive regulators. For instance, miRNA let-7 promotes tumorigenesis by regulation of KRAS and NRAS transcripts [52]. miRNAs are known to regulate pathways controlled by genes such as p53, MYC, and RAS. Furthermore, miR17-92 cluster has been shown to be able to act as a functional switch between cell proliferation and apoptosis. miRNAs have also been shown to be involved in apoptosis. For example, some miRNAs (miR-497, miR-128, miR-15, and miR-16) can induce apoptosis by targeting BCL2 in neuronal cells [9, 53, 54]. miR-21 can reduce apoptosis by targeting PDCD4 proapoptotic gene that has been linked to Alzheimer's disease [18, 32, 55]. Interestingly, PDCD4 3′ UTR also contains a conserved response element for miR-144 suggesting that the aging-specific miR-144 might play a role in the inhibition of apoptosis by repressing the activity of PDCD4. In addition, knockdown of Dicer has been shown to result in increased cortical apoptosis and causes neuronal dysfunction and degeneration [17, 56].

### 4.2. Cell Cycle Regulation

Cell cycle progression can be halted by two separate but interacting pathways the p53 and p16-pRb pathways. Our current knowledge remains sketchy about the kind of damage that may cause a neuronal cell to undergo apoptosis or senescence under different stimuli. It is likely that the effect of intacellular and environmental factors is both cell type as well as stimulation specific. The p53 pathway mainly governs DNA damage response-induced senescence. p21 is the critical downstream transcription target of p53 and plays a key mediator role in regulating p53-induced senescence. Several miRNAs modulate the expression of p53, p21, or their downstream targets and as a consequence regulate cellular senescence [53, 54, 57].

### 4.3. Neuroinflammatory Pathways

Most acute and chronic neurodegenerative conditions are accompanied by neuroinflammation, yet the exact nature of the inflammatory processes and whether they are trigger or consequence of disease progression is not well understood. Various cellular mechanisms associated with neurodegeneration are activated or enhanced by inflammatory processes that may contribute to mitochondrial dysfunction, oxidative stress, or apoptosis of neurons. More recently, tumor necrosis factor (TNF) and interleukin-1-beta (IL-1*β*) signaling pathways have been found to play a role in the pathogenesis of Alzheimer's and Parkinson's diseases [10, 55]. TNF signaling plays a key role in mediating neuronal cell death, and TNF receptor deficiency appears to have a neuroprotective effect [55]. IL-1*β* overexpression has been implicated as factor in the initiation and progression of Alzheimer's disease [58]. Moreover, IL-1*β* promotes *APP* transcription and translation in various cell types [59–61]. miR-101 reduced APP expression after prolonged IL-1*β* treatment, suggesting a role for miR-101 in the control of APP expression in response to IL-1*β* in Alzheimer's disease [10].

### 4.4. Ubiquitin-Proteasome Pathway

The accumulation of misfolded proteins is a recurring event during brain aging and is exacerbated in several neurodegenerative diseases, including SCA1 and Alzheimer's diseases [62–64]. It has been suggested that protein accumulation may result from a dysfunction in the ubiquitin proteasome system (UPS). Indeed, there is mounting genetic and biochemical evidence of an involvement of the ubiquitin proteasome system in SCA1 [65, 66]. We have recently identified HECTD1 and RNF8 E3 ubiquitin-protein ligases as targets of ncRNA in the cortex and cerebellum of individuals diagnosed with spinocerebellar ataxia type 1 and Alzheimer's disease (Persengiev et al., submitted). The HECT family of protein ligases ubiquitinate proteins for degradation by the 26S proteosome protein complex and have nonredundant functions in regulating specific signaling cascades [67, 68]. As such, deregulation of HECT ligases and the miRNAs that regulate their expression can severely perturb neuronal structure and function and may lead to functional collapse of the postmitotic neurons and withdrawal from the brain circuitry.

### 4.5. Insulin/IGF Pathway

The interacting pathways of the insulin/insulin-like growth factor (IGF) pathway, target of rapamycin (TOR) pathway, and sirtuin family are all involved in regulatory networks controlling the food intake that impact generally on longevity, and miRNAs are involved in the regulation of each pathway. The role of miRNAs in regulating these pathways and their significance for organism aging has been comprehensively discussed in a recent paper by Baek et al. [25].

To summarize, the IGF and TOR pathways are conserved and well-defined regulatory signaling pathways, which play a critical role in protein synthesis and glucose homeostasis. Mutations that inhibit these pathways or cause expression reduction are known to extend lifespan in *C. elegans* [69], *Drosophila* [70], mice [8], and even humans [71, 72], and evidence from lower organisms has revealed the significance of miRNAs in modulating the IGF pathway. For example, it has been reported that overexpression of miR-100 inhibits both mTOR mRNA and protein levels [73]. Sirtuins promote longevity by reducing calorie intake in yeast and mammalian cells [74]. SIRT1 was linked to brain physiology and neurological disorders. In a mouse model of Alzheimer's disease, the induction of SIRT1 expression improved neuronal survival and suppressed *β*-amyloid production [75, 76]. SIRT1 exerts the neuroprotective effect by activating the retinoic acid receptor beta that leads to induction of *α*-secretase (ADAM10). ADAM10 in turn inhibits *β*-amyloid production. In addition, ADAM10 activation by SIRT1 also induces the Notch signaling pathway, which is known to repair neuronal damage in the brain [76]. miR-217 is progressively increased during ageing in the endothelial cells, and it can reduce SIRT1 expression through binding to a cognate response elements within SIRT1 3′ UTR of SIRT1 [77]. miR-34a is a downstream target of p53, and it also targets SIRT1 [56], indicating a connection between SIRT1 and the ageing signaling pathway.

## 5. Concluding Remarks

**Figure 3:**
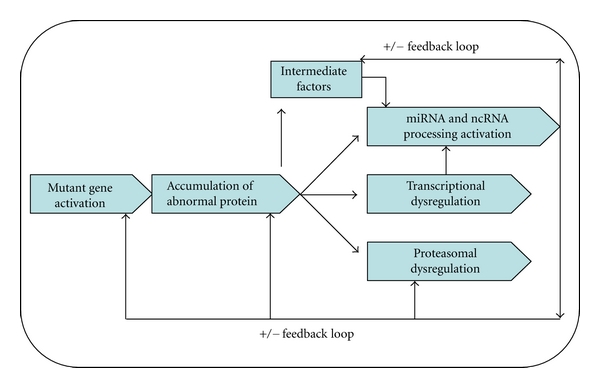
Model depicting miRNAs regulatory networks in the brain compartments affected by neurodegenerative disorders and the potential protective feedback loops that operate during aging-induced functional deterioration.

The discovery of miRNAs has revealed a new layer of regulation of gene expression, and studies in recent years have shown that miRNAs not only have a unique expression profile in the brain and peripheral nervous system but also play crucial roles in the regulation of both neuronal cell development and function. miRNA play an important role in the molecular control of brain development and subsequently in the aging process and associated neuron pathologies. The complex networks affected by miRNAs and the regulatory feedback loops are summarized in Figure 3. Even though the precise situation is likely considerably more complicated, these studies provide a new insight into how the cell regulatory systems, for example, interactions between miRNAs, cell signaling, and transcription pathways, are involved in multiple cellular activities that influence brain aging.

In this paper, we summarized the data that reveal a new role for miRNAs in brain aging and neurodegeneration. These studies not only demonstrate that miRNA-mediated inhibition is important for maintaining neuron homeostasis, but also they show that release of this inhibition can be an important part of increased brain susceptibility to external and internal stress during the aging process. Protein synthesis has long been identified as essential for the formation of long-term memories, and it is therefore not entirely surprising that miRNAs are involved in this process.

The field of miRNA and ncRNA research has developed quickly, and with the identification of brain-specific miRNAs in recent years, a new level of understanding of brain abnormalities associated with the aging has been acquired. However, more work remains to be done to fully understand the miRNA mechanism of action in normal brain aging and neurodegenerative conditions, so that expression of the miRNAs can potentially be exploited as a new point of entry for therapy. With the growing number of miRNAs and ncRNAs, each carrying a long list of putative targets, the challenge is now to annotate their biological functions.

## Figures and Tables

**Table 1:** List of miRNAs that are reported or have high probability to inhibit the expression of genes linked to neurodegenerative disease development.

Disease	Risk gene	miRNA	References
Alzheimer's disease	A-beta	miR-101	[10]
Parkinson's disease	*α*-synuclein	miR-7, miR-153	[11]
Spinocerebellar ataxia type 1 (SCA1)	Ataxin 1	miR-144, miR-107, miR-130, miR-19	[12, 13]
Amyotrophic lateral sclerosis (ALS)	SOD1	miR-206	[14]

Spinocerebellar ataxia type 7 (SCA7)	Ataxin 7	miR-199*, miR-141*, miR-200a*	[15, 16]
Huntington's disease	Huntingtin	miR-216*, miR-107*, miR-27ab*, miR-128*	[15, 16]

*miRNAs that have high potential to inhibit ataxin 7 and Huntingtin gene expression as predicted by Target Scan (release 5.2).
